# Two case studies of very long-term retention

**DOI:** 10.3758/s13423-021-02002-y

**Published:** 2021-09-28

**Authors:** Ashleigh M. Maxcey, Richard M. Shiffrin, Denis Cousineau, Richard C. Atkinson

**Affiliations:** 1 Department of Psychology, Vanderbilt University, Nashville, TN 37212 USA; 2 Department of Psychological Brain Sciences, Indiana University, IN 47405 Bloomington, USA; 3 Brain and Mind Research Institute, University of Ottawa, ON K1N 6N5 Ottawa, Canada; 4 Department of Psychology, University of California, CA 92093 San Diego, USA

**Keywords:** Long-term memory, Permastore, Permanence of memory, Skill retention, Memory for skilled search

## Abstract

Here, we present two case studies of extremely long-term retention. In the first, Richard C. Atkinson (RCA) had learned word sequences during experiments for his dissertation. Sixty-seven years later, RCA relearned the same words either in the original order or in a scrambled order. RCA reported no conscious awareness that the words were those used in the dissertation, but his relearning was considerably better for the words in the original order. In the second case study, Denis Cousineau had searched displays of objects for the presence of a target. The targets and foils had been novel at the beginning of training, and his search rate improved markedly over about 70 sessions. After 22 years, retraining showed retention of much of this gain in rate of search, and the rate was markedly faster than search for new objects with the same structure as the trained set. We consider interpretations of these case studies for our understanding of long-term retention.

**Supplementary Information:**

The online version contains supplementary material available at 10.3758/s13423-021-02002-y.

It is common for memory theorists to distinguish short-term and long-term memories and to assume that the long-term memories are very long-lived(e.g., Atkinson & Shiffrin, 1968). Failures of long-term memory are assumed to be due to retrieval failures (e.g., Shiffrin, 1970) and to processes such as context change and interference (e.g., McGeoch, 1932). Modest support for extremely long retention comes from a variety of case studies in which material is originally well learned, is arguably unrehearsed or unretrieved over long subsequent periods, and is then shown to exhibit clear evidence of retention. Examples include Bahrick (1984), Bahrick et al. (1975), Bahrick and Hall (1991), Conway et al. (1991), Squire (1989), and Stanhope et al. (1993). These examples include retention of Spanish and of the names and faces of high school classmates. As is the case in most such studies, and in the present report, it is difficult to know whether any relevant exposures have occurred during the delay period. In these examples, though, reasonable conjectures about the kinds of exposure can be made.

Here we report two more examples of long-term retention: retention over 67 years of word sequences, demonstrated by better relearning of words in the originally learned order than in a scrambled order, and retention over 22 years of an ability to search displays for what were initially novel objects. The present findings certainly cannot demonstrate that long-term memory is permanent, given that many memories cannot be retrieved after long delays, and given that memories can be modified by retrieval events occurring after original learning (e.g., Loftus et al., 1978; Loftus & Palmer, 1974). Demonstrations like the present ones, however, in which originally learned procedures or memories are unlikely to have been used or retrieved for very many years, lend some credence to theories holding that failures of long-term memory for events or procedures that have not been activated for long periods are due to failures of retrieval of records that still exist in memory.

## Case Study 1: Memory after 67 years

Our first case study is of author Richard C. Atkinson (RCA). While conducting experiments for his PhD dissertation in 1954 (Atkinson, 1954, 1957), he learned fixed sequences of adjectives that he used to train 84 participants in an anticipation serial learning procedure. Prompted by a request to retrieve his dissertation from the Indiana University archives, RCA wondered if he still retained any of that learning, and whether it might be possible to demonstrate such retention.

The original task involved anticipation learning (Hull et al., 1940). The words were presented one at a time every 2 seconds with an apparatus known as the “Hull drum.” As each word was presented, the participant tried to call out the next word. To score the participants’ verbal responses in real time, RCA had to have complete mastery of the list.

The decision was made to use relearning to test retention rather than, say, recognition, because relearning has been shown to be a more sensitive test of retention. Studies comparing relearning of studied stimulus pairs with learning of those stimuli in new pairings include those of Groninger and Groninger (1980), MacLeod (1988), and Nelson (1978). Richard M. Shiffrin (RMS) and Ashleigh M. Maxcey (AMM) adapted these procedures for relearning using an anticipation method.

RCA learned two new lists comprised of samples of words from his dissertation: half of each list had words in the original order, and the other half had words in a scrambled order. Verbal responses were recorded and then scored offline. In one learning session (on one day) the list consisted of 12 adjectives and the old order was first; in the other learning session (on another day) the list consisted of 14 adjectives and the scrambled order was first. The words first encountered during each relearning session had an advantage, so we report average performance across the two sessions.

RCA was blind to the experimental manipulation. He presumed (incorrectly) that some of the tested words would be from the original list, whereas other words would not be part of the original list. He reported no awareness during relearning that any of the words tested were from his dissertation. RCA’s comments were made following testing during informal conversation with AMM, who did not  confirm or deny RCA's impressions.

We report these data on relearning as anecdotal but suggestive, with results joining a number of other reports showing very long-term retention. We do not think it worthwhile to report statistics because such a small sample could have been a chance occurrence, however defined. Nonetheless, we see the data as worth reporting because scientists do not plan experiments on memory with 67-year retention intervals.

More details of the procedures and analyses, and additional discussion, are provided in the Supplementary Information (SI).

### Results

**Fig. 1:**
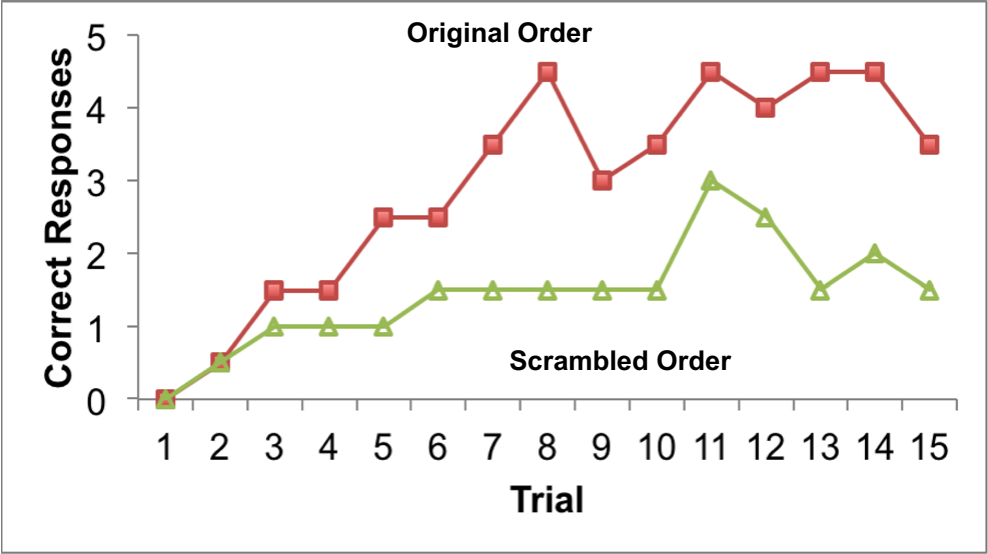
Memory after 67 Years. Correct anticipations across 15 learning trials for words presented in the original order (closed red squares) and words presented in a scrambled order (open green triangles). Responses are averaged over 2 days of learning, across which order condition was counterbalanced. (Color figure online)

Figure 1 illustrates the number of correct anticipations made for the half of the list presented in the original order (closed red squares) and for the half of the list presented in scrambled order as a function of the learning trial (open green triangles). The first cycle through the list was the initial exposure to the list, and thus there are no data to report for Trial 1. The results are averaged across 2 days in which the orders of the first and second halves of the list were counterbalanced. Performance generally improved across 15 cycles through each list. The relearning of the original order (a grand average of 2.9 correct words) was superior to the relearning of the scrambled order (a grand average of 1.6 correct words) performance being almost twice as good for the original order. Although these results cannot be claimed to be reliable given the circumstances, they do provide evidence for retention of word associations across a 67-year interval.

### Discussion

Although the relearning results should be considered “suggestive,” it is nonetheless worth speculating how relearning might have been facilitated after such a long time. RCA reported no awareness that any of the words relearned had any special status, or even that they were words used in his dissertation. That lack of awareness could be due in part to the context used for relearning: The relearning context certainly differed substantially from that present during original learning. In addition, the words used in the dissertation certainly must have been encountered in numerous different contexts in the 67 years since, so that any feeling of “familiarity” that might ordinarily produce a sense of recognition of these words would be lost due to massive retroactive interference.

What, then, could account for improved relearning of words in the original order? The words in question were adjectives such as *angry, precise, timid,* and so on. In the 67-year retention interval, these adjectives might have been seen in immediate succession, in either the original or a new order, only on relatively rare occasions. Thus, the sequential relations among the words (i.e., associative memory) might have been spared from the retroactive interference that would have interfered with memory for individual words (i.e., item memory; Cox & Criss, 2017; Cox et al., 2018; Criss & Shiffrin, 2004). We did not test for recognition memory for successive words in original or scrambled order but based on the general lack of awareness that the words were those from the dissertation and due to the shift of context between the 1950s and 2021, we suspect that such tests would not have shown above-chance performance. The advantage of relearning is that it should be less dependent on context matching than is the case for overt recognition (Groninger & Groninger, 1980; Nelson, 1978).

## Case Study 2: Memory after 22 years

This case study involved author Denis Cousineau (DC), one of several participants in a visual search study that took place in 1998–1999. In that study, DC searched for the presence of a target (50% of the trials had one target) in displays of sizes 1, 2, and 4, doing so in a variety of conditions over 74 one-hour sessions. The stimuli were initially novel (circles with four spokes extending outward; see the SI): Four stimuli were always targets, and four were always foils throughout training. Some of the results were published in 2004 (Cousineau & Shiffrin, 2004), in 2015 (Cousineau, Donkin, & Dumesnil, 2015), and additional results from one of the conditions were published in 2021 (Harding et al., 2021). DC had thus seen the stimuli on a few occasions in the years from 1999 to 2021, but had not practiced search. In the study reported here, after 22 years, DC carried out 15 sessions of training in the basic conditions of the original study with the same stimuli. Retraining with the original stimuli was followed by an additional 15 search sessions that used a novel set of stimuli having the same structure (see the SI for the stimuli and a detailed report of the results and analyses).

### Results

**Fig. 2:**
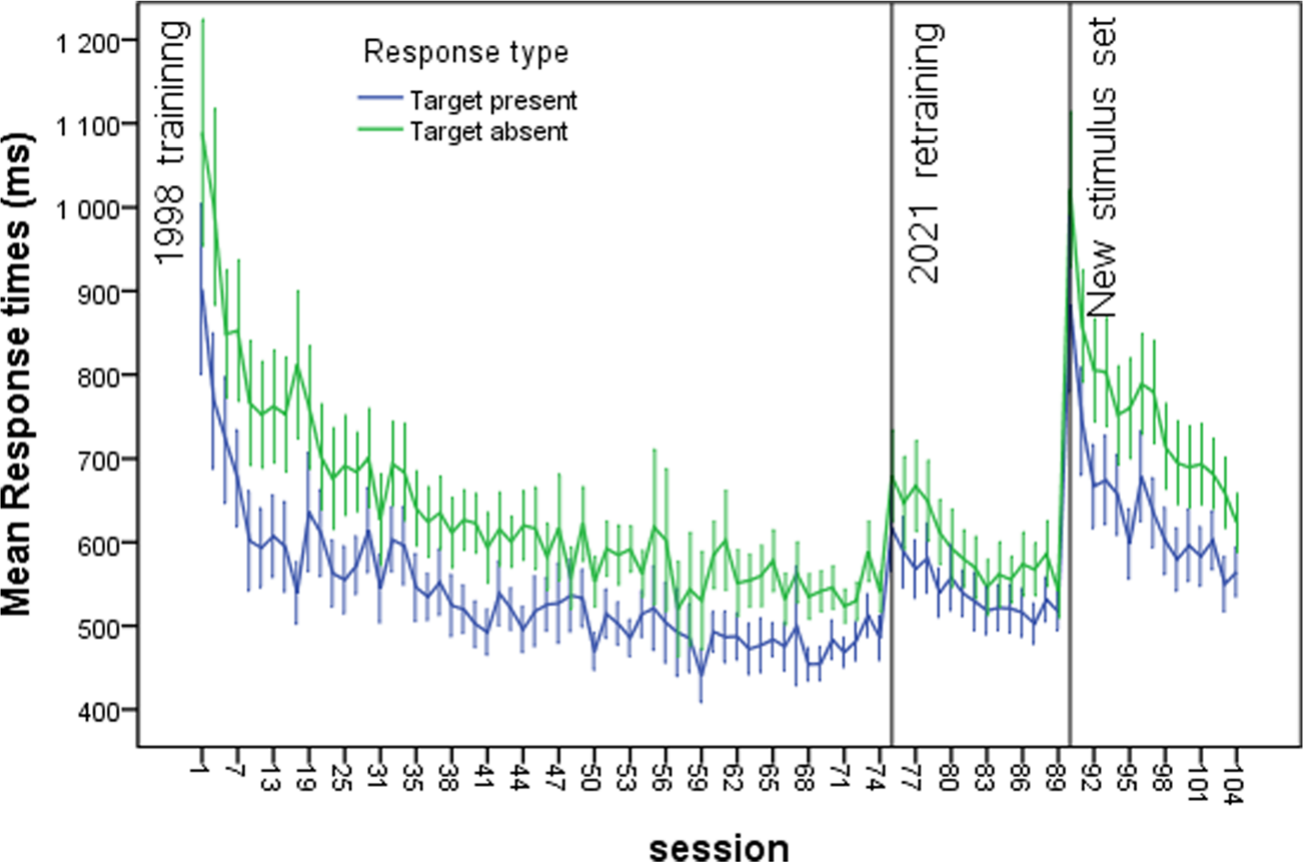
Mean response time (RT) per session for target present (blue) and target absent (green) responses along with 95% confidence intervals of the mean. (Color figure online)

**Fig. 3:**
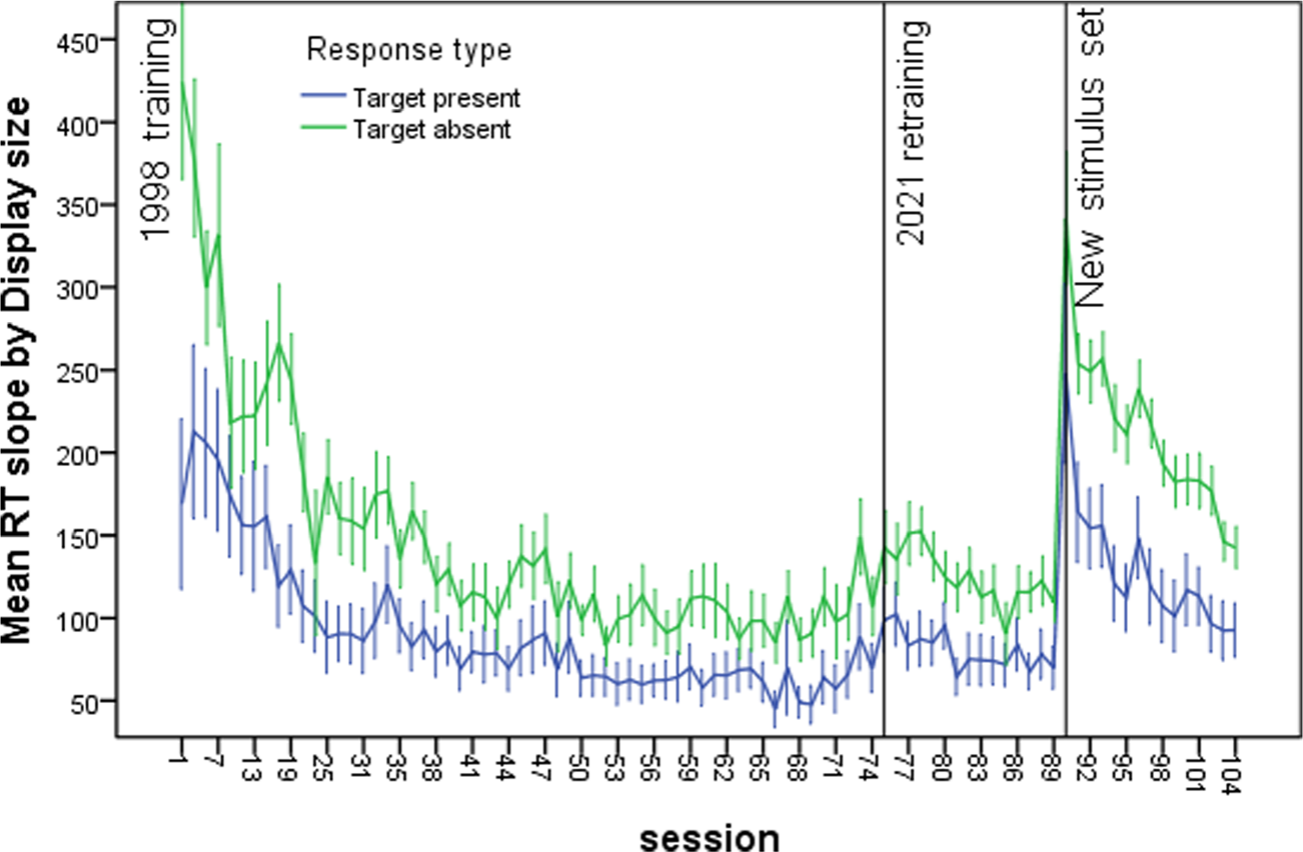
Rates of search by session of training, for original learning, for relearning after 22 years, and for learning new stimuli after 22 years. Rates are measured by the slope of response time by set size. Error bars are 95% confidence intervals of the mean. (Color figure online)

Both in the original study and in retraining, accuracy was extremely high, and was also very high for the training on the new stimuli, in all cases well above 90%. Consequently, we focus here on the response times. The results of primary interest are shown in Figs. 2 and 3, showing mean RTs per session and average slope values (mean RT as a function of set size).

The results may be summarized by noting that transfer of original learning across 22 years was excellent. For mean RTs, the starting level after 22 years was about at the level of the 12th search session in original learning (for the first 35 sessions, the odd sessions were used for an unrelated task—see the SI), and rapidly approached the final asymptotic level in original learning. For slopes, the starting level was close to the final level of original learning. For the new stimuli, new search learning was required, demonstrated both by mean RTs and slopes, although a general benefit was seen compared with original learning, probably due to general task learning—“learning to learn” (see Postman, 1970)—rather than the processes of search per se.

### Discussion

In the intervening 22 years, DC had on occasion viewed the original stimuli when producing new publications but had not practiced search with them, and certainly had not encountered them in everyday life. He reported high degrees of recognition and feelings of familiarity for them both prior to and during retraining. He had not carried out visual search of any kind remotely similar to the usual laboratory procedures during the 22-year retention interval, probably contributing to a lack of interference for the procedures of search. There have been reports of substantial retention of motor skills for long periods (e.g., Adams, 1987; Hikosaka et al., 2002), although whether accompanied with awareness has not been settled (Corkin, 1968; Willingham & Dumas, 1997), and long-term retention of implicit learning even in Alzheimer’s patients (Knopman, 1991).^1^ Motor learning in the form of eye movements may have played a role in initial learning by DC, but the speed of search became fast enough that eye movements, if they were taking place, likely lagged behind the processes of search, comparison, and identification.

The processes of search that likely were learned and retained include general task learning independent of the stimuli. The response times, however, probably included some processes that are not retained but need to be learned on each new occasion, so that retention measured by response times would not have been perfect. On the other hand, the processes of search are unlikely to have seen interference during the retention period. These processes are well measured by search slopes, and show almost perfect retention for the original stimuli. There are several learning processes that determine the slopes, as laid out by Schneider and Shiffrin (1977), Shiffrin and Schneider (1977), and Shiffrin and Lightfoot (1997). Search for novel stimuli likely begins by serial comparisons of each visual stimulus to each member of the target set, and each comparison may occur feature by feature. As shown by Shiffrin and Lightfoot (among other research), with experience the stimuli become unitized and comparisons can occur in a single step, rather than by features. As shown in the 1977 publications, two other types of learning likely took place, one in which the four target items became a category that could be searched in a single step, and another by which a target in the display could attract attention automatically (on some trials) so that the first comparison was the target. The slope results suggest excellent retention of each of these learned processes.

## General discussion

When event records and procedures are subsequently retrieved, they are invariably modified and altered by additional storage occurring at the time of each subsequent retrieval (e.g., Loftus et al., 1978; Loftus & Palmer, 1974). Thus, investigating long-term memory permanence of such records and procedures is best restricted to cases for which the original memory is unlikely to have been retrieved during the retention interval. That is very likely the case for the two studies reported here. These studies are of additional interest due to the long retention intervals involved, especially that of 67 years.

The present results and others like them show that at least some memories can survive for extremely long periods. Of course, these results do not speak to the permanence of all long-term memory records. They do lend mild support to the hypothesis that long-term memories are permanent to a surprising degree when left undisturbed (obviously excepting sufficiently severe neural degradation). Such a hypothesis is probably untestable and unverifiable, because retrieval after long time periods will often fail for a variety of reasons, particularly due to change of context between original learning and later test. That it can succeed to the extent illustrated here is, however, impressive.

## Supplementary Information

ESM 1 (DOCX 5409 kb)

## Footnotes

1 Author R. M. Shiffrin informally noted good retention of the skill of bicycle riding when, due to the pandemic, he took up this activity again after 65 years.
