# Supplementary material for: Two case studies of very long-term retention
Source: Psychon Bull Rev. 2021 Sep 28;29(2):563–7. doi: 10.3758/s13423-021-02002-y (PMC9038803; doi:10.3758/s13423-021-02002-y)
Supplement: Supplementary file 1 — (DOCX 5409 kb) [file 13423_2021_2002_MOESM1_ESM.docx]

**Two case studies of very long-term retention**

# Supplemental Material

# Case Study 1: Memory after 67 years

The 1954 dissertation (Atkinson, 1954; Atkinson, 1957) reported anticipation learning by 84 participants for lists of varying lengths and intertrial intervals using the same words in the same order. The lists were composed of two-syllable adjectives of five to seven letters, no words repeating across lists. While conducting his dissertation, Richard C. Atkinson (RCA) scored the responses by hand in real time. At the time of relearning in 2021, RCA recalled that he had learned the sequential order of the lists extremely well, though reported no conscious awareness of the words that had been used through informal conversations with RMS and AMM.

One of the originally learned lists was 12 items long. In the current study, the words on this list were used for relearning on Day 1. The first six words were in the original order, the next six words in a scrambled order (i.e., no successive words in the original order). Anticipation learning continued for 15 passes through the lists. Another of the originally learned lists was 14 words long. The words on this list were used for relearning on Day 2. The first seven words were in a scrambled order and the last seven in the original order. Again, learning proceeded for 15 passes through the list. On both days, each word was presented for 6 seconds during which time RCA attempted to produce the word to follow. There was a 10-second break after each of the 15 cycles through the list. The 10 seconds were tracked by ten small circles in the center of the screen, one of which disappeared every second to denote a countdown to the start of the next trial. On Day 1, the 10-second countdown was followed by a 4-second ‘ready’ signal (i.e., a green fixation cross). The ready signal was removed on Day 2 because the experiment was running effectively, rendering the ready signal unnecessary.

**Original Order**

**Figure A1**. Relearning data for Day 1. Correct anticipations across 15 learning trials for words presented in the **original order (red, closed squares)** and words presented in a **scrambled order (green, open triangles)**.


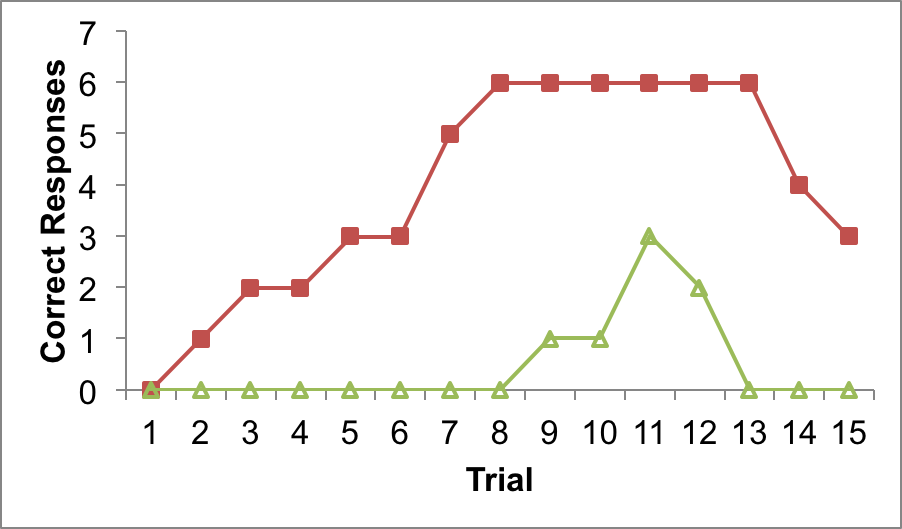


The 12 words on the first list were angry, precise, timid, uphill, normal, flanking, dirty, certain, mixed, gentle, stylish, and lower. The 14 words on the second list were direct, random, sudden, brittle, yearly, unseen, graphic, vicious, intact, classic, active, tardy, hollow, and extreme.

**Scrambled Order**

The experiment was coded in cognition.run and shared via Zoom by Ashleigh M. Maxcey (AMM), located in Nashville, TN, with Richard C. Atkinson, located in La Jolla, CA, simulating the Hull task (Hull et al., 1940) previously used by RCA (Atkinson, 1954; Atkinson, 1957). During the experiment, AMM was muted with her camera off and RCA only viewed the experiment on his screen. RCA’s verbal responses were recorded using Zoom and scored offline.

*Open-data procedure.* Links to the cognition.run experiments and a PDF of the 1954 dissertation can be found on Open Science Framework at https://osf.io/wxgmk/?view_only=00c51ea125eb4bdc81cbf919e60ebc36.

**Scrambled Order**

**Original Order**


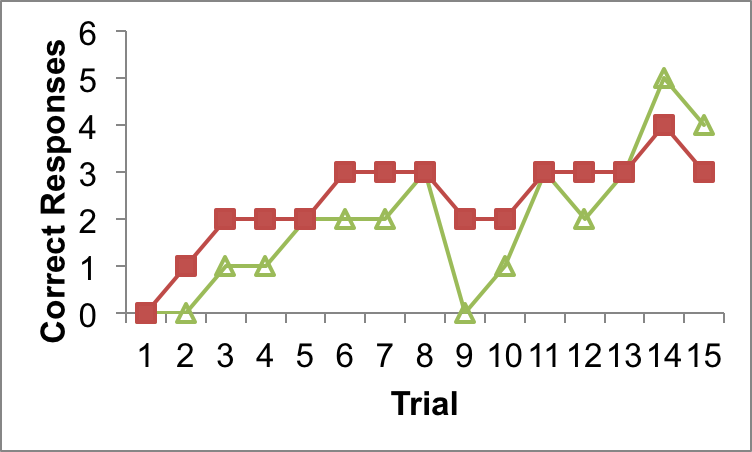


**Figure A2**. Relearning data for Day 2. Correct anticipations across 15 learning trials for words presented in the **original order (red, closed squares)** and words presented in a **scrambled order (green, open triangles)**.

**Results**

**Figures A1** and **A2** show the relearning data for Days 1 and 2 respectively. It is clear that the words first encountered on each day had a considerable advantage, so the main text reports the average of the two days. The two lists in relearning differed slightly in length, so we could have instead reported probability of recall each day and then averaged those probabilities in the main text. That way of reporting in no way changes the pattern of findings.

It is interesting that RCA was unaware that the manipulation used in relearning was one of order. Although he reported no conscious awareness that any of the words being relearned were those from his dissertation, he did suspect that the manipulation involved both old words from the dissertation and new words. When informed of the order manipulation at the end of the experiment, he expressed surprise. At the end of the experiment, RCA was disturbed and apologetic for performing (in his words) so poorly. In retrospect, he now believes that the scrambled parts of the list disrupted the overall learning process.

# Case Study 2: Memory after 22 years

Denis Cousineau (DC) was one of a few subjects who carried out the original visual search study in 1998/9. He acted as a subject for more than 70 sessions of about an hour in length. The study was very complex with many conditions and only a summary of the most relevant aspects will be reported here. Additional details can be found in Cousineau and Shiffrin (2004). The participant labeled C in that report is DC.

*Apparatus.* The new sessions were programmed in E-Prime 2.0 (Schneider et al., 2012) and presented on a LCD screen (60 Hz refresh rate). The apparatus mimicked the original experiment, including stimulus presentation and the response keys.

**Figure A3.** The stimuli used in the experiment. The stimuli on the left were originally used in 1998 and were reused in the relearning phase. The stimuli on the right served as the new stimuli used in sessions 90 to 104. In each stimulus set, the first four are targets and the last four were foils. In the original 1998 stimulus set, the critical features are the spokes radiating away from the inner circle. In the new stimulus set, the critical features are the inner squares. In the 1998 stimulus set, the diagnostic features represent the clock position 10:30 or 4:00. In the 2021 stimulus set, the diagnostic squares are on locations (row, columns) (1,1) and (3,2) or (2,1) and (4,1).

*Stimuli.* The white stimuli (26 pixels wide × 50 pixels tall) were presented on a black background (**Fig. A3)** in a dark room. They were shown using VGA resolution (640 × 480 pixels, each stimulus sustained slightly less than 1**°** when seen at a distance of 60 cm).

*Procedure.* The task involved searching for one of four possible targets among a display containing one, two, or four items. On half the trials, a target was present and the correct response was to press the ‘1’ key on the numeric keypad with the right forefinger. When no target was present, the correct response was ‘2’ with the right middle finger.

A trial began with the presentation of a center fixation star for 1,000 ms, followed by indicators of the positions that the stimuli on the current trial were going to occupy. The original and retraining study used the outline circles (without spokes) as indicators. The new stimuli used the outline rectangles (without internal squares) as indicators. The indicators lasted for 1000 ms. Then the search stimuli were shown until response or for a maximum of 15 s.

Different trials used different presentation schedules for the timing of the display objects and object features; these trials were mixed pseudorandomly. Condition 1, 33%: All objects and features were displayed simultaneously. Condition 2, 33%: All trial’s object positions were displayed simultaneously, but the features arrived one at a time, cumulatively; the rates of feature arrivals were at ISIs of 16.66 ms, 33.33 ms, or 50.00 ms, with equal probabilities. On half of the target present trials, the two features diagnostic for a correct decision were shown first; on the other half of the target present trials, those two features were shown last. Condition 3, 33%: The display objects, with all features present, were shown one at a time at ISIs of 16.66 ms, 33.33 ms, or 50.00 ms, with equal probabilities. In half of the target present trials, the target was the first object shown; in the other half of the target present trials, the target was the last object shown. The original study and retraining with the old and new stimuli used this procedure.

In the original study, the even sessions through session 34 used an unrelated task, not a search task, and the results from those sessions are not reported in the figures in the main text, nor in **Figure A4** here (these results are reported in Cousineau et al. (2015)). Thus, the figures compress the scale labeled ‘session’ until session 35, allowing appropriate comparison to the retraining sessions.

*Open-data procedure.* The experiment used the *Born-open for E-prime* package (Cousineau, 2020). Data were automatically backed up on GitHub at the end of every session. The experiment, stimuli, raw data, preliminary analysis scripts, and additional analyses can be found at <https://github.com/VIC-Laboratory-ExperimentalData/RSP-22yearsLater>.


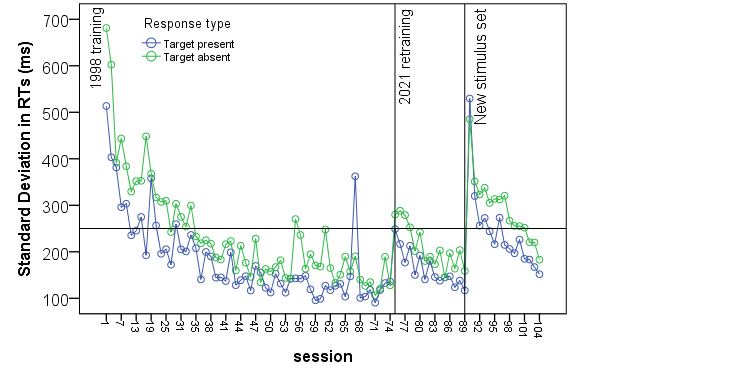


**Figure A4**: Standard deviation of response times by session.

**Results**

For this report, the results are cumulated across all the timing conditions, showing cumulated results for target present and target absent separately. The reported analyses focus on the response time results because accuracy in all cases was well above 90%. The main text gives the summary results for mean response times and slopes. A variety of other analyses show the same patterns of findings. To give one example, the variability of the response times is shown in **Figure A4**.

For a variety of reasons, one would expect slopes to be a better indicator of retention than raw response times. In particular, massive retroactive interference likely occurred for processes used for motor responses on the keyboard, because DC used keyboards extensively during the retention interval, but not with special emphasis on the two keys used for task responding.

The slopes reflect the rate of search through the displays; these rates should not be much affected by increases or decreases in the speed of pressing the two response keys. As reported in the main text, the slopes show a very high degree of transfer of learning for the original stimuli. The new stimuli show a decrease in slopes over sessions roughly in line with the decrease in slopes in original learning, suggesting the learning of the same processes at the same rates as those learned in the original study (see the main text for discussion of the processes that might have been learned). The slopes at the very outset of training for the new stimuli were somewhat lower than at the very outset of the original study (the slopes for the new stimuli started at about the level seen in the original study at the 2^nd^ to the 4^th^ session, suggesting some nonspecific task learning that occurs over a few sessions that is somewhat independent of the stimuli used).

Many people have experiences in everyday life that make them aware that procedural learning and motor skills are retained well for periods of years without practice. Thus, the retention of search skills by DC is probably less surprising than the retention of word sequences by RCA, even though the skill of searching is mostly a cognitive rather than a motor skill. It is of course possible that the degree of retention of memories varies for motor skills, cognitive procedures, language skills and episodes from life. It is also possible that the differences across such domains are due to different amounts of retroactive and proactive interference, context change, and other factors known to affect retrieval. Present knowledge does not provide answers to these questions.
